# Using a Delphi consensus process to develop an acupuncture treatment protocol by consensus for women undergoing Assisted Reproductive Technology (ART) treatment

**DOI:** 10.1186/1472-6882-12-88

**Published:** 2012-07-07

**Authors:** Caroline A Smith, Suzanne Grant, Jane Lyttleton, Suzanne Cochrane

**Affiliations:** 1 Centre for Complementary Medicine Research, University of Western Sydney, Locked Bag 1797, Penrith South DC, NSW, 2751, Australia; 2 Jane Lyttleton, Paddington Medical Centre, 198 Oxford St, Paddington, NSW, 2021, Australia; 3 Suzanne Cochrane, School of Health and Science, University of Western Sydney, Locked Bag 1797, Penrith South DC, NSW, 2751, Australia

## Abstract

**Background:**

Assisted reproductive technologies (ART) are increasingly utilised for resolving difficulties conceiving. These technologies are expensive to both the public purse and the individual consumers. Acupuncture is widely used as an adjunct to ART with indications that it may assist reducing the time to conception and increasing live birth rates. Heterogeneity is high between treatment protocols.

The aim of this study was to examine what fertility acupuncturists consider key components of best practice acupuncture during an ART cycle, and to establish an acupuncture protocol by consensus.

**Methods:**

Fifteen international acupuncturists with extensive experience treating women during ART interventions participated in 3 rounds of Delphi questionnaires. The first round focused on identifying the parameters of acupuncture treatment as adjunct to ART, the second round evaluated statements derived from the earlier round, and the third evaluated specific parameters for a proposed trial protocol. Consensus was defined as greater than 80% agreement.

**Results:**

Significant agreement was achieved on the parameters of best practice acupuncture, including an acupuncture protocol suitable for future research. Study participants confirmed the importance of needling aspects relating to the dose of acupuncture, the therapeutic relationship, tailoring treatment to the individual, and the role of co-interventions. From two rounds of the Delphi a consensus was achieved on seven treatment parameters for the design of the acupuncture treatment to be used in a clinical trial of acupuncture as an adjunct to ART. The treatment protocol includes the use of the traditional Chinese medicine acupuncture, use of manual acupuncture, a first treatment administered between day 6–8 of the stimulated ART cycle which is individualised to the participant, two treatments will be administered on the day of embryo transfer, and will include points SP8, SP10, LR3, ST29, CV4, and post transfer include: GV20, KD3, ST36, SP6, and PC6. Auricular points Shenmen and Zigong will be used. Practitioner intent or *yi* will be addressed in the treatment protocol.

**Conclusions:**

Despite a lack of homogeneity in the research and clinical literature on ART and acupuncture, a consensus amongst experts on key components of a best practice treatment protocol was possible. Such consensus offers guidance for further research.

## Background

There are increasing numbers of women reporting delays with conceiving. In Australia one in six women report infertility and, 50% seek fertility services [1]. Over the last five years, the number of assisted reproductive technology (ART) procedures has increased on average by 10% per year in Australia, despite the significant personal costs of ART [2]. In fact, Australia has one of the highest levels of utilisation at 1,574 cycles per million population, with the average cost of a standard in vitro fertilization (IVF) cycle at $5,492. ART is a resource intensive and costly treatment option for women and their families, and the Government, and new therapies that improve reproductive and health outcomes are highly desirable.

Acupuncture is an emerging and widely used therapy as an adjunct to IVF, and it is important to consumers, and health care providers that the evidence base for this therapy be established. Randomised controlled trials, and several systematic reviews and meta-analyses have examined whether acupuncture as an adjunct to ART treatment improves reproductive outcomes. The first two reviews reported a benefit from acupuncture in improving reproductive outcomes[3],[4]. Stener-Victorin and Manheimer commenting on these early reviews and a further four meta-analyses, concluded that the evidence that acupuncture improves clinical pregnancies or live birth rates is unclear due to significant heterogeneity between trials [5]. This relates to both clinical and methodological heterogeneity such as the use of different study questions, use of controls ranging from usual care alone to invasive and non invasive procedures, and use of different treatment protocols.

The treatment protocols used in many of the studies examining the use of acupuncture as an adjunct to embryo transfer (ET) vary between the acupuncture points selected, the frequency and duration of treatment, mode of stimulation, depth of needle insertion, and the role of co-interventions. This may be influenced by the different styles or traditions of acupuncture including traditional and classical acupuncture, auricular acupuncture, trigger point acupuncture, and single point acupuncture. Traditional Chinese Medicine (TCM) and classical Acupuncture are based on theoretical concepts of Yin and Yang and the Five Elements, and explain disease and physiological function. A westernised medical application of acupuncture involves the use of acupuncture using trigger points, segmental points and commonly used formula points. Medical acupuncture may involve the application of acupuncture based on the principles of neurophysiology and anatomy. Auricular therapy involves the use of the ear to make a diagnosis and subsequent needling to points on the ear.

There is debate in the acupuncture community whether the acupuncture performed in these randomised controlled trials reflects the practice of real world acupuncture [6]. Often no diagnostic framework is applied, and there is a lack of individualisation to address specific TCM imbalances and symptoms, and the dose of acupuncture used is described as low [6]. It has also been proposed that the rationale for some points was difficult to understand and justify, for example the use of acupuncture points Spleen 6 and Large intestine 4 are contra-indicated in pregnancy [7]. Limitations identified in previous research highlight the need for future clinical trials to reflect acupuncture in real world clinical practice and thereby increase clinical validity.

To date there has been no attempt at determining what might constitute an adequate acupuncture protocol for the treatment of women undergoing a cycle of ART in a clinical trial. In 2011 we received funding to conduct a randomised controlled trial evaluating the effect of acupuncture on live birth rates for women undergoing IVF. It was recommended that the trial evaluate a treatment based on best practice achieved through consensus. The aim of this study was to examine what experts in fertility acupuncture consider key aspects and components of best practice acupuncture during an ART cycle, and to establish an acupuncture protocol by consensus.

## Methods

Consensus methods are often used to select and reduce items, and are usually designed to combine the knowledge and expertise of experts. We used the Delphi technique to address our objectives. The Delphi method is based on a structured process for collecting and distilling knowledge from a group of experts by means of a series of questionnaires interspersed with controlled opinion feedback [8]^.^ It was also suitable due to the number of participants we wanted to involve, the written procedure, the anonymity of the comments, and the time available to conduct our study.

The research study was approved by the University of Western Sydney Human Research and Ethics Committee, and conducted at the Centre for Complementary Medicine Research at the University of Western Sydney.

### Selection of participants

Twenty eight English speaking international and national acupuncturists working in the fertility area were invited to participate in the Delphi process. The criteria used to identify potential participants included a history of publishing on acupuncture and fertility, or previous experience of having undertaken or currently undertaking research on acupuncture and fertility, or a minimum of ten years experience of using acupuncture treatment for fertility enhancement or sub-fertility and consider themselves specialists in fertility acupuncture; or recommended from participants meeting the above criteria.

### Procedure

#### Selection of items

For the development of the initial item pool parameters were identified through reports and guidelines as contributing to the parameters of acupuncture treatment [9-11]. These were presented as follows:

a) treatment as an adjunct to the ART cycle; including the acupuncture rationale (style of acupuncture), extent to which treatment is varied, timing of acupuncture treatment during the time an ART cycle, use of preferred acupuncture points at this time (identified from a review of literature and textbooks), needling technique, needling stimulation and retention, patient education, the role of the practitioner-patient relationship,

b) treatment as an adjunct to embryo transfer; including treatment frequency, use of specific acupuncture points, the use of co-interventions, practitioner intent, location of where treatment should be administered, and

c) demographics of participants including age, gender, acupuncture qualification, years in practise, workload attributed to working with fertility patients, and style of acupuncture practised.

Participants were emailed or posted an invitation to participate in the Delphi process. For those agreeing to participate a link to an online survey hosted at http://www.surveymonkey.com was provided. Two (2) electronic reminders were sent.

During the first round participants were asked to indicate their opinion about acupuncture treatment parameters for women receiving acupuncture as an adjunct to ART. Responses were indicated by yes or no, and by ranking items along a four point Likert scale for agreement (“strongly agree”, “agree”, “disagree”, “strongly disagree”). We also included an opportunity for participants to comment on their responses, or to provide additional comments on other components of treatment (Additional file 1). The aim of this first round was to identify what fertility acupuncturists considered key components for women during an ART cycle. Following analysis of the first round, a summary report was circulated to participants on the results from the first round, and they were invited to participate in the second round.

For the second round, participants were asked to similarly respond to statements. An agreement of 80% or more was required to achieve a consensus to retain an item from those responding to each statement. Three rounds only were undertaken to achieve consensus. During this third round a series of statements were presented to participants describing the parameters of the proposed treatment protocol for our study, we sought an 80% agreement (from those responding to each statement) to achieve a consensus.

### Analysis

Data were collated by http://www.surveymonkey.com, and responses were downloaded both as summaries and detailed comments. The responses to each item were reported as the proportion of participants. CS and SG discussed the qualitative and quantitative responses after round one, and circulated the results of round one and two to participants. CS and JL met to discuss the items circulated for the consensus statement describing the treatment protocol for the trial treatment protocol.

## Results

Of the 28 people invited to participate 17 agreed to participate, and 17 completed round one, 16 completed round two and 15 completed round three. The characteristics of the Delphi participants are described in Table 1. Two participants joined the study on the basis of recommendations from other participants.

**Table 1:** Profile of responders participating in the Delphi process

***Characteristic (n = 17)***	**% n**
*Gender*	
Male	11.8 (2)
Female	82.3 (14)
Missing	5.9 (1)
*Age*	
26–35	23.5 (4)
36–45	23.5 (4)
46–65	53.0 (9)
*Highest TCM qualification*	
Graduate diploma	17.6 (3)
Bachelors degree	17.6 (3)
Masters degree	29.4 (5)
PhD	29.4 (5)
Other	5.9 (1)
*Years in acupuncture practice*	
6–10	47.1 (8)
11–20	29.4 (5)
21+ years	23.5 (4)
*Country of Practice*	
Australia	35.3 (6)
United States	23.5 (4)
China	11.8 (2)
Denmark	5.9 (1)
Sweden	5.9 (1)
United Kingdom	5.9 (1)
Missing	11.8 (2)
*Styles of acupuncture practiced (multiple responses given)*	
TCM	82.3 (14)
Classical acupuncture	35.3 (6)
Western Medical	17.6 (3)
Five element	23.5 (4)
Auricular	17.6 (3)
Other (e.g.Japanese)	23.5 (4)
*Acupuncture patient seen/week*	
0–35	35.3 (6)
36–50	29.4 (5)
51+	23.5 (4)
Not currently in practice	11.8 (2)
*Percentage patient consults for fertility related treatment*	
26–50%	11.8 (2)
51–75%	11.8 (2)
76–100%	64.7 (11)
Not currently in practice	11.8 (2)
*Patients undergoing IVF* majority of client base	5.9 (1)
50% of client base	41.1 (7)
25% client base	29.4 (5)
Not in practice	23.6 (4)

### First Round of Delphi: Key aspects and components of acupuncture during an ART cycle

The frequency, number of treatments and timing of acupuncture during an ART cycle were identified as important. In particular, treatments administered early during the stimulation phase of the cycle, and treatment on the day of ET (Table 2). Participants indicated that the need for treatment was influenced by the individual’s presentation, and that frequent treatments were administered early on during the ART cycle with the goal to improve follicular and endometrial responsiveness. Participants’ responses ranged from a minimum of twice weekly treatments to every other day up until the time of the trigger injection. Eight suggested there be one treatment within the week following ET.

**Table 2:** Round 1: Acupuncture treatment parameters for women undergoing ART

**Timing of acupuncture in relation to an ART cycle**	**Yes % n = 17**
Day of ET	94.1 (16)
During the stimulation phase	94.1 (16)
Days 3–5 between egg pick up and ET	53.0 (9)
Following ET but before pregnancy test	47.1 (8)
Following trigger but before egg retrieval	41.1 (7)
Day of egg retrieval	35.3 (6)
Day of trigger	17.6 (3)
Needle stimulation and retention	
Important to attain deqi	82.4 (3)
Maintain deqi	
No need to stimulate again	47.1 (8)
Stimulate after 10 min	23.5 (4)
Stimulate after 15 min	17.6 (3)
Deqi not important	5.9 (1)
Mode of stimulation	
Manual and electro-acupuncture	53.0 (9)
Manual only	35.3 (6)
Electo-acupuncture only	0.0 (0)

Participants selected from a list of ‘essential points’ those to be selected for use during the stimulation phase of the ART cycle. Channels selected included the Ren, Kidney, Stomach, Liver and Spleen channels, with points on the innervation area to the uterus and ovaries in the abdominal muscles and in the leg, and the use of extra points. The most common points included Ren 4 (Guanyuan) and 6 (Qihai), Kidney 3 (Taixi), Spleen 6 (Sanyinjiao), Zigong, Stomach 36 (Zusanli), and Stomach 29 (Guilai). The use of ‘essential points’ during other stages of the ART cycle was less clear.

Fourteen participants indicated a needling duration of 25–30 minutes. The majority of participants (15) responded that obtaining *deqi* was important (Table 2). Views varied on the need to maintain *deqi* during treatment with eight of the 17 responders reporting there was no need to stimulate again. Nine responders suggested use of combined application electro and manual acupuncture.

Advice on self care was frequently given and included rest, regular sleep, not working excessive hours, minimising stress, dietary advice, keeping positive, the need to relax, no vigorous exercise, avoiding extreme heat or cold, no heavy lifting, avoiding fumes and cleaning fluids and general advice explaining the rationale for acupuncture at this time.

The practitioner-patient relationship was identified as important, and comments describing features of this included; ensuring practitioners were present, positive, vital, reassuring, empathetic and calm and confident without conveying unrealistic expectations. A suggestion was also made to the importance of practitioners having sound knowledge and understanding of reproductive physiology and pathology.

Nine participants identified practitioner intent or *yi* as important. Some participants described their intent as:

"“my intention is never to ‘get a patient pregnant’ during ART.It is instead to optimise their response, minimise side effects and to support them emotionally so they can be at peace with either a negative or positive response”."

"“to relax them and allow them to feel empowered by what they are doing to assist the IVF procedure”."

"“to let go of outcome, and let go of ego, I try to avoid attachment to outcome because I feel it blocks the flow of Qi through me in my treatments, and prevents me from visualising the meridian pathways effectively”."

### Treatment as an adjunct to embryo transfer

The majority of participants considered TCM (n = 13), and classical acupuncture (n = 9) as the best style of acupuncture for this treatment. Others suggested western medical acupuncture (n = 4), five element (n = 5), Japanese (n = 3) and auricular styles (n = 6) Over half (n = 10) of participants thought a semi standardised protocol should be used (some fixed treatment options and some individualisation), with five out of 17 responders advocating for use of a completely individualised protocol. Eleven responders thought three of more treatments should be administered to achieve a successful implantation and that the administration of acupuncture should be at a minimal distance from the ART clinic. Preference was for a location within 30 minutes of the ART clinic (n = 8), and five considered treatment should be on the premises of the ART clinic. The role of co-interventions was seen as important with moxa (n = 5) and herbs (n = 5) the most commonly cited interventions.

For treatments administered on the day of ET, two participants reported essential points as described in the Paulus protocol [12], other participants nominated other essential points. Only two participants thought auricular acupuncture was not important to a treatment protocol.

### Second round of Delphi

In the second round of the Delphi process, we sought to identify areas for consensus relating to our own future trial, and more generally to guide future research. Participants were asked to give their response along a scale indicating their disagreement or agreement with a statement describing aspects on the intervention. Resourcing for our trial allowed for treatment on the day of ET and one additional treatment, questions focused on gaining consensus for style, timing and acupuncture points for this treatment protocol.

Among those responding to the statement there was over 80% agreement that TCM (n = 15), classical acupuncture (n = 12), and auricular acupuncture (n = 13) were suitable for our clinical trial. Fourteen of the 16 responders agreed that the protocol should be semi-standardised, incorporating some fixed treatment components and some individualisation. Seven responders expressed a first preference for the first treatment to be administered on day 8 of the ART cycle (around the time of scan and bloods), five expressed a second preference for day 3 (start of stimulation), and seven expressed a third preference for day 12 (trigger injection).

Between one and four participants chose not to respond to the question seeking their opinion on the acupuncture points to be used in the first treatment. Among those responding eighty percent agreement was obtained on the use of the following acupuncture points : innervation areas close to ovaries/uterus, PC6 (Neiguan), LR3 (Taichong), ST29 (Zusanli), Zigong (located 3 cun lateral to the midline), Ren4 (Guanyuan), Ren6 (Qihai), ST36 (Zusanli), SP6 (Sanyinjiao), SP8 (Diji), SP10 (Xuehai), Yintang, KI3 (Taixi), KI13 (Qixue), Bl23 (Shenshu), HT7 (Shenman), and Chongmai & Renmai (Table 3).

**Table 3:** Round 2: selection of acupuncture points during the stimulation phase of the cycle

**Acupuncture points Responders n = 16**	**Strongly agree % (n)**	**Agree % (n)**	**Disagree % (n)**	**Strongly disagree % (n)**	**Missing Data % n**
Point selection individualised	25.0 (4)	37.5 (6)	18.7 (3)	0.0	18.7 (3)
Innervation areas close to ovaries/uterus	62.5 (10)	18.7 (3)	12.5 (2)	0.0	6.2 (1)
PC6 (Neiguan)	12.5 (2)	75.0 (12)	0.0	0.0	12.5 (2)
LR3 (Taichong)	12.5 (2)	75.0 (12)	0.0	0.0	12.5 (2)
LR5 (Ligou)	0.0	25.0 (4)	50.0 (8)	0.0	25.0 (4)
ST29 (Guilai)	25.0 (4)	75.0 (10)	0.0	0.0	12.5 (2)
Zigong	56.2 (9)	25.0 (4)	6.2 (1)	0.0	12.5 (2)
ST30 (Qichong)	0.0	43.7 (7)	31.2 (5)	0.0	25.0 (4)
Ren4 (Guanyuan)	50.0 (8)	37.5 (6)	0.0	0.0	12.5 (2)
Ren3 ((Zhongji)	25.0 (4)	31.2 (5)	18.7 (3)	0.0	25.0 (4)
Ren6 (Qihai)	25.0 (4)	50.0 (8)	6.2 (1)	0.0	18.7 (3)
ST36 (Zusanli)	37.5 (6)	43.7 (7)	0.0	0.0	18.7 (3)
SP6 (Sanyinjiao)	37.5 (6)	37.5 (6)	0.0	0.0	25.0 (4)
SP8 (Diji)	6.2 (1)	62.5 (10)	12.5 (2)	0.0	18.7 (3)
SP10 (Xuehai)	6.2 (1)	75.0 (12)	0.0	0.0	18.7 (3)
Yintang	18.7 (3)	62.5 (10)	6.2 (1)	0.0	12.5 (2)
KI3 (Taixi)	25.0 (4)	56.2 (9)	0.0	0.0	18.7 (3)
KI12 (Dahe)	12.5 (2)	43.7 (7)	18.7 (3)	0.0	25.0 (4)
KI13 (Qixue)	12.5 (2)	50.0 (8)	12.5(2)	0.0	25.0 (4)
KI16 (Huangshu)	0.0 (0)	25.0 (4)	43.7 (7)	0.0	31.2(5)
BL23 (Shenshu)	25.0 (4)	50.0 (8)	12.5 (2)	0.0	12.5 (2)
Bl32 (Ciliao)	6.2 (1)	56.2 (9)	18.7 (3)	0.0	18.7 (3)
HT7 (Shenmen)	0.0 (0)	68.7 (11)	12.5 (2)	0.0	18.7 (3)
Chongmai & Renmai	56.2 (9)	18.7 (3)	6.2 (1)	0.0	18.7 (3)

The response to the selection of acupuncture points on the day of embryo transfer was almost 100%. Eighty percent of participants agreed the following points should be considered for inclusion in the treatment protocol administered on the day of ET (Table 4). These included; PC6 (Neiguan), Yintang, HT7 (Shenman), SP8 (Diji), LR3 (Taichong), ST29 (Guilai), ST36 (Zusanli), SP6 (Sanyinjiao), SP10 (Xuehai), KI3 (Taixi) and Du20 (Baihui). The majority of participants recommended the timing of these points to be:

Before ET only; SP8 (Diji), LR3 (Taichong), ST29 (Guilai), SP6 (Sanyinjiao),

After ET only; ST36 (Zusanli), SP10 (Xuehai), KI3 (Taixi), Du20 (Baihui)

Suitable for before and/or after; PC6 (Neiguan), YinTang, HT7 (Shenmen)

Fourteen participants agreed that as the points HT7 (Shenmen), PC6 (Neiguan) and Yintang have similar functions, the acupuncturist should select the point best suited to the individual. A manual mode of stimulation was considered most appropriate by 10 participants, with five suggesting a combination of manual and electro-acupuncture was acceptable for use at this time. Twelve responders agreed *deqi* should be maintained for acupuncture administered during the stimulation phase, and 7 agreed *deqi* should be maintained with additional stimulation during treatment before ET but not after ET. Two auricular points were considered important for consideration in the treatment protocol, these included Shenmen (n = 14), and Zigong (n = 12).

**Table 4:** Round 2: selection of acupuncture points for treatment on the day of embryo transfer

**Acupuncture points Responders n = 16**	**Strongly agree % (n)**	**Agree % (n)**	**Disagree % (n)**	**Strongly disagree % (n)**	**Missing % (n)**
Innervation areas close to ovaries/uterus	18.8 (3)	25.0 (4)	56.3 (9)	0.0	0.0 (0)
PC6 (Neiguan)	43.8 (7)	56.3 (9)	0.0	0.0	0.0 (0)
LR3 (Taichong)	37.5 (6)	56.3 (9)	6.3 (1)	0.0	0.0 (0)
ST29 (Guilai)	37.5 (6)	50.0 (8)	12.5 (2)	0.0	0.0 (0)
ST30 (Qichong)	6.3 (1)	37.5 (6)	50.0 (8)	6.3 (1)	0.0 (0)
Ren4 (Guanyuan)	43.8 (7)	31.3 (5)	25.0 (4)	0.0	0.0 (0)
Ren6 (Qihai)	20.0 (3)	40.0 (6)	40.0 (6)	0.0	6.3 (1)
ST36 (Zusanli)	43.8 (7)	43.8 (7)	6.3 (1)	6.3 (1)	0.0 (0)
SP6 (Sanyinjiao)	37.5 (6)	56.3 (9)	0.0	6.3 (1)	0.0 (0)
SP8 (Diji)	25.0 (4)	56.3 (9)	18.8 (3)	0.0	0.0 (0)
SP10 (Xuehai)	18.8 (3)	62.5 (10)	12.5 (2)	6.3 (1)	0.0 (0)
Yintang	43.8 (7)	43.8 (7)	12.5 (2)	0.0	0.0 (0)
KI3 (Taixi)	31.3 (5)	50.0 (8)	18.8 (3)	0.0	0.0 (0)
HT7 Shenmen)	18.8 (3)	62.5 (10)	18.8 (3)	0.0	0.0 (0)
KI13 (Qixue)	12.5 (2)	50.0 (8)	37.5 (6)	0.0	0.0 (0)
SP4 (Gongsun)	12.5 (2)	37.5 (6)	43.8 (7)	6.3 (1)	0.0 (0)
LI 4 (Hegu)	18.8 (3)	56.3 (9)	12.5 (2)	12.5 (2)	0.0 (0)
Bl32 (Ciliao)	12.5 (2)	37.5 (6)	43.8 (7)	6.3 (1)	0.0 (0)
Du20 (Baihui)	37.5 (6)	62.5 (10)	0.0	0.0	0.0 (0)

### Other treatment related components: the role of the practitioner

Many participants in round one commented on the importance of practitioner intent or *“yi”*. In round two all participants agreed this meant the practitioner being calm, being prepared for both negative and positive outcomes (n = 15), being non judgemental (n = 14), establishing good rapport with the patient (n = 14) and being present (n = 13).

### Role of co-interventions

All participants recommended providing advice to all patients to minimise stress. Relaxation techniques were identified as important and approaches recommended by participants for women to use included; meditation (n = 13), yoga (n = 12), visualisation (n = 8), or tai chi (n = 7).

Other advice considered appropriate for some women depending on their presentation included the need to keep warm and avoid cold environments (14 participants) Avoiding stimulants such as caffeine was considered important by 15 participants, 14 suggested avoiding Spleen depleting foods, cold foods, and vigorous exercise. The use of positive visualisations was seen as important by 12 out of 16 participants.

### Future research ideas

The areas identified as important to acupuncture treatment in round one and two emphasise the role of acupuncture as a complex intervention in clinical practice. There is a need for future research to consider pragmatic research designs for women undergoing ART. Participants suggested research on the following interventions; moxa (auricular therapy (n = 8), herbs), relaxation techniques and dietary therapy (In addition, participants suggested the following areas relating to dosing studies for future research of acupuncture administered during ART, electro-acupuncture versus manual acupuncture, and the role of moxibustion.

### Round 3

Based on the findings from Round 2 the proposed protocol for our clinical trial was presented to participants who were requested to vote if they agreed or disagreed with the components:

· A TCM rationale will be used for the trial.

· Manual acupuncture would be administered during an ART cycle including ET, and would be semi-standardised.

· The first treatment will be administered between day 6–8 of the stimulated ART cycle, and will include core points ST29, CV4, CV6, SP6, SP10, plus up to 5 individualised additional points based on TCM pattern differentiation.

· Treatment on the day of ET will consist of treatment pre and post transfer. Points to be used for pre transfer include SP8, SP10, LR3, ST29, CV4 and one selected from HT7/PC6/YinTang (depending on presentation of women). Points to be used post transfer include: GV20, KD3, ST36, SP6, and PC6.

· Auricular acupuncture using points Shenmen and Zigong only will be used on the day of ET.

· *De qi* will be maintained with additional stimulation during the initial treatment on day 6–8, and during the pre embryo treatment.

· Practitioner intent or *yi* will be addressed in the treatment protocol.

Agreement of 86% was obtained from 15 people completing Round 3.

## Discussion

The aim of this study was to examine what fertility acupuncturists consider key components of best practice acupuncture during an ART cycle, and to establish an acupuncture protocol by consensus for women using ART, and on the day of embryo transfer for use in a clinical trial. The study participants confirmed the importance of needling aspects relating to the dose of acupuncture, the therapeutic relationship, tailoring treatment to the individual, and the role of co-interventions. From two rounds of the Delphi process a consensus was achieved on seven treatment parameters for a protocol to be used in a clinical trial.

Our findings reinforce our understanding of acupuncture as a complex intervention, and participant feedback from working with this clinical group of patients highlighted the relevance and particular importance of the non needling aspects of care and treatment. Individualisation of care was reflected through participant’s responses relating to treatment during the stimulation phase of the ART cycle, the selection of acupuncture points, tailoring of advice on self care and use of co-interventions to improve follicular and endometrial responsiveness and manage stress. The importance of the practitioner-patient relationship was acknowledged by the thoughtful contributions and suggestions from participants particularly during the initial exploratory questions relating to the practitioners intention, or *yi.* Clearly *yi* was much more than establishing rapport through warmth and friendliness, but the preparation and nurturing of intent when healing. Participation by a diverse group of practitioners and acupuncture researchers has enabled us to develop a treatment protocol to address the research question for which funding has now been received. The treatment protocol defines the characteristics of the needling processes to be used, incorporates flexibility during the initial treatment to tailor treatment to the individual, and to address aspects of the therapeutic relationship.

Acupuncture is increasingly being used by women seeking fertility support, with evidence on the use of acupuncture for infertility support informed by two surveys. Firstly, data from 9,408 patients consulting acupuncturists identified 1.3% as using acupuncture for fertility support [13]. Secondly, a survey of UK acupuncturists indicates 15% provide fertility support, and for some practitioners this has become a large proportion of their caseload [14]. Eighty percent reported most of this work was related to assisted conception, and 20% of practitioners indicated additional benefits to their patients including reduced stress levels, and improved relaxation as benefits from acupuncture. The additional benefits to patients from acupuncture were similarly highlighted by our group of practitioners and researchers.

The external validity of our treatment protocol is supported by similarities with the acupuncture treatment protocol developed for a pragmatic design evaluating the role of acupuncture for women experiencing delays with conceiving in a primary care setting [15]. In this study a process of consensus building for the trial protocol emphasised the high value placed on a differential diagnosis, and the importance of the therapeutic relationship in working towards the therapeutic outcome.

The strengths of this study include the comprehensive approach taken to identifying the dimensions for consideration relating to this area of clinical practice. We also achieved consensus from experienced practising acupuncturists from this area of clinical practice, and active researchers contributing to examining the evidence base for acupuncture. There are several limitations to this study. Not all items explored in round one were explored further in the subsequent rounds, this may reflect the diversity of acupuncture styles practiced by our Delphi participants, and the differing emphasis given to different items. There was some missing data to most items, and it was unclear for some items why this occurred. The characteristics of our responders indicate they were experienced practitioners, or researchers in terms of qualifications and the focus of their clinical practice with seeking fertility support. Their acupuncture training and experience will have influenced their responses to the Delphi rounds, and although we asked responders the style of acupuncture they use to treat women, we do not know if this reflected their initial training. Other limitations could relate to the participation of experts. We set out to be inclusive, however not all who were invited accepted our invitation, and there may be other styles of acupuncture practiced that were not included, or were under-represented. Our methodology did not consistently allow us the explore the reasons for participants responses to some questions. For example we do not know the rationale for participants indicating their agreement or disagreement with selected acupuncture points. This study offers no insight to some practitioner concerns about the use of points such as Spleen 6 in pregnancy, or in the pre-implantation phase.

A limitation of this study has implications for future research directions. Although the majority of study participants gave agreement to the treatment protocol we would use in our trial there was concern about the low dosage of acupuncture to be used. A view was shared that future protocols should be more pragmatic and in particular allow a greater number of treatments to be administered over a longer period of time, and allow treatments to be individualised. The problem of low dose or sub optimal use of acupuncture used in clinical trials has also been highlighted by several authors. White et al. [16], highlight the importance of defining an adequate dose of acupuncture using a neurobiological; approach, and more recently in the Langevin, et al., [17]*.* Areas for future research for acupuncture and treatment of individuals with infertility or sub-fertility proposed by our study participants concur with some of the directions highlighted in the article *Paradoxes in Acupuncture Research: Strategies for Moving Forward* article which identified the apparent disconnect between experimental results and clinical trial results in acupuncture research [17]*.*

## Conclusion

This study has addressed a need to ensure research in this area of acupuncture practice is based on a consensus. It is intended these characteristics of acupuncture form the foundation of our clinical protocol, and that these findings can positively influence the directions of future acupuncture research area is this growing area of practice.

## Competing interests

The authors declare that they have no competing interests. No funding was obtained for this study.

## Authors’ contributions

CS conceived and designed the study, undertook analysis and interpretation of data. SG contributed to the design, conducted the study and contributed to the analysis of data. JL contributed to the interpretation of data. SC contributed to the design of the study. All authors helped draft the manuscript, and read and approved the final manuscript.

## Pre-publication history

The pre-publication history for this paper can be accessed here:

http://www.biomedcentral.com/1472-6882/12/88/prepub

## Supplementary Material

Additional file 1 Delphi Forum on Acupuncture for Women undergoing ART: Round 1. Click here for file
