# Supplementary material for: Using a Delphi consensus process to develop an acupuncture treatment protocol by consensus for women undergoing Assisted Reproductive Technology (ART) treatment
Source: BMC Complement Altern Med. 2012 Jul 7;12:88. doi: 10.1186/1472-6882-12-88 (PMC3416745; doi:10.1186/1472-6882-12-88)
Supplement: Additional file 1 — Delphi Forum on Acupuncture for Women undergoing ART: Round 1. [file 1472-6882-12-88-S1.docx]

**Additional File 1.**

**Delphi Forum on Acupuncture for Women undergoing ART: Round 1**

1. Please indicate the style of acupuncture you consider is best for women receiving acupuncture around the time of embryo transfer?

*Traditional Chinese medicine (TCM)*

*Classical acupuncture*

*Western medical*

*Five element*

*Japanese*

*Korean*

*Auricular*

1. For women undergoing an embryo transfer and receiving acupuncture, do you think the treatment protocol should be (choose one of the following).

*Individualised*

*Semi standardised (some fixed treatment components, and some individualisation)*

*All standardised (all fixed standardised components*

1. How many treatments do you think are needed around the time of embryo transfer to achieve a successful implantation?

*1*

*2*

*3*

*4*

*5+*

1. The following questions refer to the timing of acupuncture treatments.

*Yes No*

1. *During the stimulation phase of the cycle*
2. *Day of trigger*
3. *Following trigger but before egg retrieval*
4. *Day of egg retrieval*
5. *Days 3-5 between egg pick up and embryo transfer*
6. *Day of embryo transfer*
7. *Days following transfer but before pregnancy test*

*If you answered yes to any of the above, please give details of how many treatments and when*

1. For the treatments you said yes to in question 4, are there essential acupuncture points you would recommend?

*Yes No*

1. *During the stimulation phase of the cycle*
2. *Day of trigger*
3. *Following trigger but before egg retrieval*
4. *Day of egg retrieval*
5. *Days 3-5 between egg pick up and embryo transfer*
6. *Day of embryo transfer*
7. *Days following transfer but before pregnancy test*
8. Thinking about needle technique, do you have any recommendations regarding the direction of needle insertion for any of the points described above?

*Yes No*

1. Do you have any recommendations regarding needle depth?

*Yes No*

1. Thinking about needle stimulation and needle retention. Considering your earlier responses what are your preferred methods of stimulation and retention (select an option from the drop down menu).

*Initial stimulation*

*Attain de qi Deqi sensation is not important*

1. Needle stimulation and retention

*Maintain de qi*

*Stimulate after Stimulate after Do not Deqi*

1. *min 15 min stimulate again sensation is not important*
2. Needle stimulation and retention

*Total retention time*

1. *in 30 min 45 min Other*
2. Needle stimulation and retention

*Manual acupuncture or electro-acupuncture*

*Manual only Electric only Combination of EA and MA*

1. Needle stimulation and retention
2. Are there any co-interventions that you consider essential to all patients around the time of embryo transfer. Please select one or more of the following.

*Herbs*

*Dietary therapy*

*Massage*

*Moxibustion*

*Other*

1. If acupuncture is performed on the day of an embryo transfer where should the acupuncture be administered?

*On the premises of the ART clinic*

*Minimal time/distance from ART clinic (please specify distance and time)*

*Time and location of no relevance*

1. Is there any information or advice you consider a patient needs that will optimise the benefit from the acupuncture treatment?
2. Do you have any specific recommendations you would like to make in relation to the patient/practitioner relationship?
3. Do you agree or disagree with the use of any of the following standardised points given just before embryo transfer?

*Strongly disagree Disagree Agree Strongly agree*

*PC-6*

*SP-8*

*LR-3*

*GV-20*

*ST-29*

*CV-4*

*CV-6*

*ST-36*

*SP-6*

*LI-4*

*KI-13*

*Yintang*

*Si Shen Cong*

1. Do you agree or disagree with the use of any of the standardised points to be given on the day after an embryo transfer?

*Strongly disagree Disagree Agree Strongly agree*

*CV-4*

*CV-6*

*ST-29*

*PC-6*

*LI-4*

*SP-10*

*ST-36*

*SP-6*

*GV-20*

*KI-3*

*Yintang*

*Si Shen Cong*

1. Auricular acupuncture has frequently been used in the published trials. What are your views on the inclusion of these auricular points in conjunction with standardised acupuncture, please indicate the importance of this to a treatment. If important indicate a selection of point(s). If you do not think it is important, select “not important at all”, and move to the next question.

*Not important at all Slightly important Important Very important*

*Shenmen*

*Zhigong*

*Neifenmi*

*Naodian*

*Retention time of auricular point*

25 min

Duration of acupuncture session

Entire day

Other

*Needles or seeds*

Stainless steel needles (0.2, 13 mm)

Ear seeds

Stainless steel ear seeds

Other

1. Are there any other components to treatment around this time that you would like to suggest?
2. Do you have any other comments about your intent or “yi” in relation to working with women at this time?
3. Can you please indicate your age range (years)

20-25

25-35

36-45

46-55

55-65

65+

1. Please indicate your gender.

Male

Female

1. Please indicate your country of birth.
2. Please indicate your country of practice.
3. Please tell us about the location of your clinic in relation to an ART clinic.

*Totally physically independent*

*Practice co-exists with ART clinic*

*Close physical proximity*

1. What is your highest acupuncture or TCM qualification?

*Diploma*

*Graduate diploma*

*Bachelor degree*

*Masters degree*

*PhD*

*Other*

1. How many years have you been practising acupuncture?

*0-5 yrs*

*6-10 yrs*

*11-15 yrs*

*16-20 yrs*

*21-25 yrs*

*25+ yrs*

1. On average, how many patients do you see a week?

*0-15*

*16-35*

*36-50*

*51-80*

*81-100*

*>100*

*Not currently in practice*

1. What percentage of these patients would you treat for fertility?

*0-25%*

*26-50%*

*51-75%*

*76-100%*

1. Please select which of the following applies most to you:

*IVF patients are 25% of my client base*

*IVF patients are 50% of my client base*

*IVF patients are the majority of my client base*

*I only treat IVF patients.*

1. What style of acupuncture do you use to support a women receiving treatment for fertility? (you may choose more than one style if you use a combination of styles).

*Traditional Chinese medicine*

*Classical acupuncture*

*Western medical*

*Five element*

*Japanese*

*Korean*

*Auricular*

**Delphi Forum on Acupuncture for Women undergoing ART: Round 2**

1. The following styles of acupuncture are appropriate for our trial

Agree Disagree

*TCM*

*Classical acupuncture*

*Auricular*

1. Acupuncture administered during an IVF cycle including on the day of an embryo transfer should be:

*Agree Disagree*

*Individualised*

*Semi-standardised (some fixed treatment components, and some individualisation)*

1. Subject to consensus, two treatments will be given on the day of embryo transfer. All participants from Round 1 agreed a treatment should be administered during the stimulation phase of the cycle. We proposed a treatment will be administered in this phase of the IVF cycle. For the trial the timing of this treatment must be standardised (although the actual treatment maybe individualised/semi standardised). Drawing upon the comments from Round 1 we have proposed the following times for this treatment. Please indicate your preference in order.

*First Second Third Fourth*

*Preference preference preference preference*

*Day 3 start of*

*Stimulation cycle*

*Day 8 around time*

*Of scan & bloods*

*Day 12 of trigger*

*Following trigger but*

*Before egg retrieval*

*Other*

1. The treatment administered during the stimulation phase maybe individualised or semi-standardised. Many participants made suggestions for acupuncture points to be used during the stimulation phase of the cycle

*Strongly agree Agree Disagree Strongly disagree*

*Point selection*

*Individualised*

*Innervation areas*

*close to uterus/ovaries*

*PC-6*

*LR-3*

*ST-29*

*Zigong*

*ST-30*

*CV-4*

*CV-3*

*ST-36*

*SP-6*

*SP-8*

*SP-10*

*Yintang*

*KI-3*

*KI-12*

*KI-12*

*KI-16*

*BL-23*

*BL-32*

*HT-7*

*Chongmai & Renmai*

Time of treatment

*Day 3 start of Day 8 around time Day 12 of Following Other*

*Stimulation cycle of scan & bloods trigger trigger but*

*before egg retrieval*

*Point selection*

*Individualised*

*Innervation areas*

*close to uterus/ovaries*

*PC-6*

*LR-3*

*LR-5*

*ST-29*

*Zigong*

*ST-30*

*CV-4*

*CV-3*

*CV-6*

*ST-36*

*SP-6*

*SP-8*

*SP-10*

*Yintang*

*KI-3*

*KI-12*

*KI-12*

*KI-16*

*BL-23*

*BL-32*

*HT-7*

*Chongmai & Renmai*

1. From Round 1 the following acupuncture points have been proposed for treatment on the day of embryo transfer, some before and some after transfer. Please indicate which points you believe should or shouldn’t be included

*Strongly agree Agree Disagree Strongly disagree*

PC-6

Yintang

HT-7

SP-8

LR-3

ST-29

CV-4

CV-6

ST-30

KI-13

ST-36

*Innervation areas*

*close to uterus/ovaries*

*SP-4*

*BL-32*

*SP-6*

*SP-10*

*LI-4*

*LI-3*

*GV-20*

*Other*

Time of treatment

*Before transfer After transfer Before and after transfer*

PC-6

Yintang

HT-7

SP-8

LR-3

ST-29

CV-4

CV-6

ST-30

KI-13

ST-36

*Innervation areas*

*close to uterus/ovaries*

*SP-4*

*BL-32*

*SP-6*

*SP-10*

*LI-4*

*LI-3*

*GV-20*

*Other*

1. Some points such as PC-6, Yintang and HT-7 have similar functions. Should the research acupuncturist administer the point best suited to that individual?

*Yes*

*No*

1. Is manual acupuncture alone acceptable?

*Yes*

*No*

*Best is a combination of manual acupuncture and electro-acupuncture*

*Electro-acupuncture alone*

1. Thinking about the treatments on the day of embryo transfer, should deqi be maintained with an additional stimulation of needles during treatment?

*Yes, in the treatment before and after embryo transfer*

*Yes in the treatment before embryo transfer but not after*

*Yes, in the treatment after embryo transfer but not before*

*No, not in any of the treatments*

1. Thinking about the treatment to be given during the stimulation phase, should deqi be maintained?

*Strongly agree*

*Agree*

*Disagree*

*Strongly disagree*

1. Auricular acupuncture has frequently been used in published trials. Please indicate the importance in conjunction with standard acupuncture. If it is important, please indicate a selection of points

*Not important at all Slightly important Important Very important*

*Shenmen*

*Zhigong*

*Neifenmi*

*Naodian*

*Needles or seeds*

*Stainless steel needles (0.2, 13 mm)*

*Ear seeds*

*Stainless steel ear seeds*

*Other*

1. Which of the following are important interventions to be investigated in future research?

*Very important Important Slightly important Not important at all*

*Auricular acupuncture vs*

*body acupuncture*

*Moxibustion vs no moxibustion*

*Electro-acupuncture vs manual acupuncture*

1. In a pragmatic trial of women undergoing ART what other modalities do you see as important to include? (Please select more than one if needed)

*Yes No*

*Herbs*

*Moxa*

*Auricular acupuncture*

*Massage*

*Dietary therapy*

*Relaxation techniques*

*Other*

1. In Round 1 many respondents noted advice that they often give to patients, please rank how important you consider the following areas and if its relevant to ALL patients or just some.

*Advice*

*Very important Important Not important*

*Keep warm*

*Avoid cold environments*

*Avoid cold foods*

*Avoid Spleen depleting foods*

*Avoid stimulants such as caffeine*

*Minimise stress*

*No vigorous exercise*

*Do fertility yoga or similar*

*Positive visualisations*

*All or some patients All patients Some patients*

*Keep warm*

*Avoid cold environments*

*Avoid cold foods*

*Avoid Spleen depleting foods*

*Avoid stimulants such as caffeine*

*Minimise stress*

*No vigorous exercise*

*Do fertility yoga or similar*

*Positive visualisations*

1. There was consensus around the need for women undergoing ART to employ relaxation techniques. Do you use or recommend any of the following techniques or approaches in addition to acupuncture to help your patient to relax?

*Yoga*

*Tai Chi*

*Visulisation*

*Hypnotherapy*

*Meditation*

1. In Round 1, many of you commented on the importance of your intent or “yi” in relation to working with women at this time, and what this meant to you. To help us better understand this, please indicate if you agree or disagree with what this means when working with women undergoing ART.

*Agree Disagree*

*Practitioner being present*

*Practitioner being calm*

*Practitioner having rapport with the patient*

*Practitioner being non judgemental*

*Practitioner being prepared for both negative and positive outcomes*

*Other*

1. Do you have any specific recommendations you would like to make in relation to the patient/practitioner relationship not covered or are there any other comments you would like to make about future research
